# Poly[[aqua­(μ-4,4′-bipyridine-κ^2^ *N*:*N*′)(μ_3_-2-nitro-5-sulfonatobenzoato-κ^3^ *O* ^1^:*O* ^1′^:*O* ^5^)copper(II)] 4,4′-bipyridine hemisolvate]

**DOI:** 10.1107/S1600536809039294

**Published:** 2009-10-03

**Authors:** Zheyu Zhang

**Affiliations:** a Department of Chemistry, Baicheng Normal College, Baicheng 137000, People’s Republic of China

## Abstract

In the title compound, [Cu(C_7_H_3_NO_7_S)(C_10_H_8_N_2_)(H_2_O)]·0.5C_10_H_8_N_2_, the Cu^II^ atom is six-coordinated by two N atoms from two different bipyridine (bipy) ligands, one sulfonate O atom and two carboxyl­ate O atoms from three 2-nitro-5-sulfonatobenzoate ligands and one water O atom in a distorted octa­hedral geometry. The bipy solvent mol­ecule lies on an inversion center. The Cu^II^ atoms are linked by the bipy ligands, forming one-dimensional chains, which are connected by the 2-nitro-5-sulfonatobenzoate ligands into a two-dimensional layer-like network. The two-dimensional structure is extended by O—H⋯O and O—H⋯N hydrogen bonds into a three-dimensional supra­molecular network.

## Related literature

For general background to copper(II) sulfonate complexes, see: Du *et al.* (2009 ▶); Li *et al.* (2009 ▶); Liu *et al.* (2009 ▶); Sonnauer & Stock (2008 ▶); Sonnauer *et al.* (2009 ▶). For related structures, see: Dong *et al.* (2009 ▶).
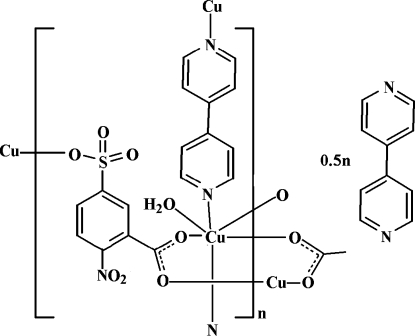


## Experimental

### 

#### Crystal data

[Cu(C_7_H_3_NO_7_S)(C_10_H_8_N_2_)(H_2_O)]·0.5C_10_H_8_N_2_
*M* *_r_* = 561.00
Monoclinic, 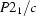

*a* = 11.4549 (17) Å
*b* = 11.0447 (16) Å
*c* = 17.089 (3) Å
β = 92.738 (3)°
*V* = 2159.5 (5) Å^3^
*Z* = 4
Mo *K*α radiation
μ = 1.17 mm^−1^
*T* = 293 K
0.23 × 0.17 × 0.14 mm

#### Data collection

Bruker SMART APEX CCD diffractometer
Absorption correction: multi-scan (*SADABS*; Bruker, 2001 ▶) *T*_min_ = 0.767, *T*_max_ = 0.850
11892 measured reflections
4260 independent reflections
2560 reflections with *I* > 2σ(*I*)
*R*_int_ = 0.088

#### Refinement

*R*[*F* ^2^ > 2σ(*F* ^2^)] = 0.065
*wR*(*F* ^2^) = 0.135
*S* = 1.00
4260 reflections
331 parameters
H atoms treated by a mixture of independent and constrained refinement
Δρ_max_ = 0.86 e Å^−3^
Δρ_min_ = −0.46 e Å^−3^

### 

Data collection: *SMART* (Bruker, 2007 ▶); cell refinement: *SAINT* (Bruker, 2007 ▶); data reduction: *SAINT*; program(s) used to solve structure: *SHELXS97* (Sheldrick, 2008 ▶); program(s) used to refine structure: *SHELXL97* (Sheldrick, 2008 ▶); molecular graphics: *SHELXTL* (Sheldrick, 2008 ▶) and *DIAMOND* (Brandenburg, 1999 ▶); software used to prepare material for publication: *SHELXTL*.

## Supplementary Material

Crystal structure: contains datablocks global, I. DOI: 10.1107/S1600536809039294/hy2230sup1.cif

Structure factors: contains datablocks I. DOI: 10.1107/S1600536809039294/hy2230Isup2.hkl

Additional supplementary materials:  crystallographic information; 3D view; checkCIF report

## Figures and Tables

**Table 1:** Selected bond lengths (Å)

Cu1—N2	1.986 (4)
Cu1—N3^i^	2.004 (4)
Cu1—O2^ii^	2.565 (4)
Cu1—O4	1.969 (3)
Cu1—O5^iii^	2.299 (4)
Cu1—O6	2.032 (4)

Symmetry codes: (i) 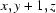; (ii) 
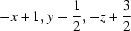
; (iii) 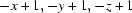.

**Table 2:** Hydrogen-bond geometry (Å, °)

*D*—H⋯*A*	*D*—H	H⋯*A*	*D*⋯*A*	*D*—H⋯*A*
O6—H1*A*⋯O1^ii^	0.83 (5)	1.94 (5)	2.758 (5)	171 (6)
O6—H1*B*⋯N4^iv^	0.86 (5)	2.00 (5)	2.801 (6)	156 (5)

Symmetry codes: (ii) 
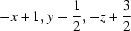
; (iv) 
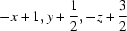
.

**Fig. 1.**
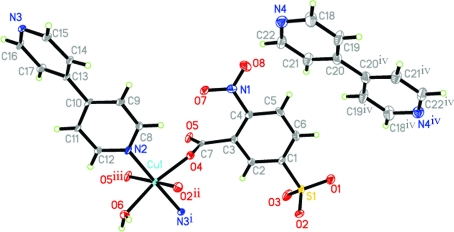
The asymmetric unit of the title compound. Displacement ellipsoids are drawn at the 30% probability level. [Symmetry codes: (i) x, 1+y, z; (ii) 1-x, -0.5+y, 1.5-z; (iii) 1-x, 1-y, 1-z; (iv) -x, 1-y, 2-z.]

**Fig. 2.**
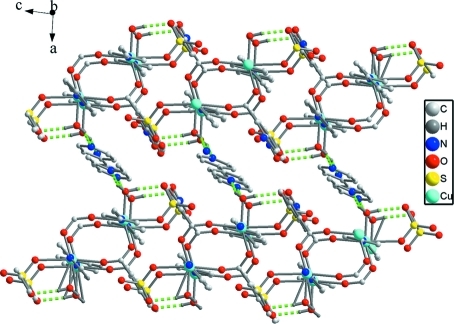
View of the three-dimensional supramolecular network in the title compound. Dashed lines denote hydrogen bonds.

## supplementary crystallographic information

### Comment

In recent years, the design and synthesis of copper(II) sulfonates have attracted great attention because of their flexible coordination modes, interesting inorganic-organic lamellar structures, selective and reversible guest inclusion properties, and their ability to intercalate guest molecules (Du *et al.*, 2009; Sonnauer *et al.*, 2009). It is noteworthy that some copper(II) sulfonate complexes with nitrogen-based secondary ligands, exhibiting different bonding modes dependent on the presence of secondary ligands, have been reported (Liu *et al.*, 2009; Sonnauer & Stock, 2008). It has also been demonstrated that the existence and changes of the secondary ligands can have a great effect on the structures of copper(II) sulfonates, often with surprising results (Li *et al.*, 2009). In this paper, we utilized 2-nitro-5-sulfobenzoic acid (H_2_nsb) as an organic sulfonate ligand and 4,4'-bipyridine (bipy) as an N-donor ligand, providing a coordination compound, [Cu(nsb)(bipy)(H_2_O)].0.5bipy, which is reported here.

In the title compound, the central Cu^II^ ion is six-coordinated by two N atoms from two different bipy ligands, one sulfonate O atom, two carboxylate O atoms from three nsb ligand and one water molecule in a distorted octahedral coordination geometry (Table 1). There are free bipy molecules in the structure, stabilized by hydrogen bonds (Fig. 1). The Cu—O distances are comparable to those found in other crystallographically characterized Cu^II^ complexes (Dong *et al.*, 2009). The Cu atoms are linked by the bipy ligands, forming an extended one-dimensional chain. These chains are further connected by the nsb ligands into a two-dimensional layer-like network. In addition, the existence of O—H···O and O—H···N hydrogen bonds (Table 2) extends the two-dimensional layer into a three-dimensional supramolecular architecture (Fig. 2).

### Experimental

A mixture of Cu(CH_3_CO_2_)_2_.2H_2_O (0.040 g, 0.2 mmol), 2-nitro-5-sulfobenzoic acid (0.049 g, 0.2 mmol), 4,4'-bipyridine (0.039 g, 0.2 mmol), and H_2_O (15 ml) was sealed in a 25 ml Teflon-lined stainless steel reactor, which was heated at 443 K for 72 h and then it was cooled to room temperature. Blue crystals of the title compound were collected.

### Refinement

H atoms on C atoms were positioned geometrically and refined as riding atoms, with C—H= 0.93 Å and *U*_iso_(H)= 1.2*U*_eq_(C). The H atoms of the water molecule were located in a difference Fourier map and refined with a distance restraint of O—H = 0.85 (1) Å and with *U*_iso_(H) = 1.5*U*_eq_(O).

### Crystal data

**Table:**

[Cu(C_7_H_3_NO_7_S)(C_10_H_8_N_2_)(H_2_O)]·0.5C_10_H_8_N_2_	*F*(000) = 1144
*M**_r_* = 561.00	*D*_x_ = 1.725 Mg m^−^^3^
Monoclinic, *P*2_1_/*c*	Mo *K*α radiation, λ = 0.71073 Å
Hall symbol: -P 2ybc	Cell parameters from 4260 reflections
*a* = 11.4549 (17) Å	θ = 1.8–26.0°
*b* = 11.0447 (16) Å	µ = 1.17 mm^−^^1^
*c* = 17.089 (3) Å	*T* = 293 K
β = 92.738 (3)°	Block, blue
*V* = 2159.5 (5) Å^3^	0.23 × 0.17 × 0.14 mm
*Z* = 4	

### Data collection

**Table:**

Bruker SMART APEX CCD diffractometer	4260 independent reflections
Radiation source: fine-focus sealed tube	2560 reflections with *I* > 2σ(*I*)
graphite	*R*_int_ = 0.088
φ and ω scans	θ_max_ = 26.0°, θ_min_ = 1.8°
Absorption correction: multi-scan (*SADABS*; Bruker, 2001)	*h* = −14→14
*T*_min_ = 0.767, *T*_max_ = 0.850	*k* = −13→13
11892 measured reflections	*l* = −21→10

### Refinement

**Table:**

Refinement on *F*^2^	Primary atom site location: structure-invariant direct methods
Least-squares matrix: full	Secondary atom site location: difference Fourier map
*R*[*F*^2^ > 2σ(*F*^2^)] = 0.065	Hydrogen site location: inferred from neighbouring sites
*wR*(*F*^2^) = 0.135	H atoms treated by a mixture of independent and constrained refinement
*S* = 1.00	*w* = 1/[σ^2^(*F*_o_^2^) + (0.0455*P*)^2^] where *P* = (*F*_o_^2^ + 2*F*_c_^2^)/3
4260 reflections	(Δ/σ)_max_ = 0.001
331 parameters	Δρ_max_ = 0.86 e Å^−^^3^
0 restraints	Δρ_min_ = −0.46 e Å^−^^3^

### Fractional atomic coordinates and isotropic or equivalent isotropic displacement parameters (Å^2^)

**Table:**

	*x*	*y*	*z*	*U*_iso_*/*U*_eq_	
C1	0.2223 (4)	0.7923 (4)	0.6944 (3)	0.0215 (13)	
C2	0.2994 (4)	0.7254 (4)	0.6511 (3)	0.0198 (12)	
H2	0.3565	0.7656	0.6243	0.024*	
C3	0.2929 (4)	0.5989 (5)	0.6472 (3)	0.0195 (12)	
C4	0.2051 (5)	0.5449 (4)	0.6866 (3)	0.0224 (13)	
C5	0.1244 (5)	0.6090 (5)	0.7293 (4)	0.0321 (15)	
H5	0.0649	0.5692	0.7542	0.039*	
C6	0.1356 (5)	0.7338 (5)	0.7335 (4)	0.0313 (15)	
H6	0.0846	0.7786	0.7627	0.038*	
C7	0.3842 (5)	0.5338 (4)	0.6006 (3)	0.0177 (12)	
C8	0.5609 (4)	0.2511 (4)	0.6504 (3)	0.0229 (13)	
H8	0.5129	0.2936	0.6831	0.027*	
C9	0.5560 (4)	0.1262 (4)	0.6501 (3)	0.0237 (13)	
H9	0.5067	0.0860	0.6831	0.028*	
C10	0.6248 (5)	0.0607 (4)	0.6005 (3)	0.0183 (13)	
C11	0.6982 (4)	0.1266 (4)	0.5532 (3)	0.0205 (13)	
H11	0.7454	0.0869	0.5186	0.025*	
C12	0.6997 (4)	0.2501 (4)	0.5582 (3)	0.0208 (13)	
H12	0.7504	0.2924	0.5273	0.025*	
C13	0.6233 (4)	−0.0732 (4)	0.5994 (3)	0.0191 (13)	
C14	0.6096 (4)	−0.1390 (4)	0.6673 (3)	0.0207 (13)	
H14	0.5960	−0.0992	0.7140	0.025*	
C15	0.6162 (5)	−0.2632 (4)	0.6656 (3)	0.0241 (13)	
H15	0.6098	−0.3057	0.7122	0.029*	
C16	0.6431 (5)	−0.2628 (4)	0.5338 (3)	0.0248 (13)	
H16	0.6541	−0.3054	0.4878	0.030*	
C17	0.6397 (5)	−0.1375 (4)	0.5304 (3)	0.0236 (13)	
H17	0.6482	−0.0971	0.4833	0.028*	
C18	0.0395 (6)	0.2255 (6)	1.0226 (4)	0.052 (2)	
H18	0.0316	0.1641	1.0593	0.063*	
C19	0.0134 (6)	0.3431 (6)	1.0445 (4)	0.0483 (19)	
H19	−0.0057	0.3597	1.0957	0.058*	
C20	0.0160 (5)	0.4360 (5)	0.9900 (4)	0.0353 (16)	
C21	0.0488 (5)	0.4042 (6)	0.9165 (4)	0.0408 (17)	
H21	0.0509	0.4629	0.8776	0.049*	
C22	0.0790 (5)	0.2855 (6)	0.8995 (4)	0.0421 (17)	
H22	0.1028	0.2677	0.8495	0.051*	
N1	0.1916 (4)	0.4121 (4)	0.6833 (3)	0.0327 (13)	
N2	0.6323 (4)	0.3136 (3)	0.6052 (3)	0.0212 (11)	
N3	0.6313 (4)	−0.3253 (4)	0.5997 (3)	0.0194 (10)	
N4	0.0754 (4)	0.1954 (5)	0.9520 (3)	0.0406 (14)	
O1	0.1331 (3)	0.9957 (3)	0.7351 (2)	0.0321 (9)	
O2	0.3416 (3)	0.9755 (3)	0.7461 (2)	0.0283 (10)	
O3	0.2451 (4)	0.9910 (3)	0.6178 (2)	0.0373 (10)	
O4	0.4740 (3)	0.5046 (3)	0.6409 (2)	0.0223 (8)	
O5	0.3643 (3)	0.5231 (3)	0.5297 (2)	0.0277 (9)	
O6	0.8116 (3)	0.4917 (4)	0.6061 (2)	0.0259 (9)	
O7	0.2618 (3)	0.3519 (3)	0.6486 (3)	0.0376 (11)	
O8	0.1112 (4)	0.3662 (4)	0.7165 (3)	0.0676 (17)	
S1	0.23675 (13)	0.95326 (12)	0.69752 (9)	0.0243 (4)	
Cu1	0.63413 (5)	0.49342 (5)	0.60435 (4)	0.0195 (2)	
H1A	0.833 (5)	0.486 (5)	0.653 (3)	0.029*	
H1B	0.843 (5)	0.543 (5)	0.576 (3)	0.029*	

### Atomic displacement parameters (Å^2^)

**Table:**

	*U*^11^	*U*^22^	*U*^33^	*U*^12^	*U*^13^	*U*^23^
C1	0.025 (3)	0.019 (3)	0.021 (3)	0.002 (2)	−0.001 (3)	−0.003 (3)
C2	0.024 (3)	0.018 (3)	0.019 (3)	−0.002 (2)	0.004 (2)	0.001 (2)
C3	0.020 (3)	0.018 (3)	0.021 (3)	0.003 (2)	0.001 (3)	−0.002 (3)
C4	0.027 (3)	0.013 (3)	0.027 (4)	0.001 (2)	0.005 (3)	0.005 (2)
C5	0.027 (3)	0.029 (3)	0.042 (4)	−0.001 (3)	0.018 (3)	0.007 (3)
C6	0.032 (3)	0.026 (3)	0.037 (4)	0.009 (3)	0.013 (3)	0.001 (3)
C7	0.030 (3)	0.007 (3)	0.016 (3)	−0.005 (2)	0.006 (3)	0.001 (2)
C8	0.029 (3)	0.015 (3)	0.027 (3)	0.000 (2)	0.014 (3)	0.001 (3)
C9	0.030 (3)	0.014 (3)	0.028 (4)	−0.004 (2)	0.007 (3)	0.002 (3)
C10	0.029 (3)	0.005 (2)	0.020 (3)	0.002 (2)	−0.007 (3)	0.001 (2)
C11	0.028 (3)	0.011 (3)	0.024 (3)	−0.001 (2)	0.010 (3)	−0.004 (2)
C12	0.026 (3)	0.011 (3)	0.027 (3)	−0.001 (2)	0.007 (3)	0.004 (2)
C13	0.024 (3)	0.009 (3)	0.025 (4)	0.000 (2)	0.005 (3)	−0.002 (3)
C14	0.030 (3)	0.013 (3)	0.021 (3)	0.002 (2)	0.010 (3)	0.000 (2)
C15	0.032 (3)	0.016 (3)	0.025 (4)	−0.001 (2)	0.008 (3)	0.005 (3)
C16	0.040 (3)	0.014 (3)	0.022 (3)	0.003 (3)	0.011 (3)	−0.006 (3)
C17	0.035 (3)	0.015 (3)	0.021 (3)	−0.001 (3)	0.006 (3)	0.006 (3)
C18	0.068 (5)	0.043 (4)	0.048 (5)	0.014 (4)	0.020 (4)	0.011 (4)
C19	0.066 (5)	0.034 (4)	0.045 (5)	0.019 (4)	0.013 (4)	−0.004 (4)
C20	0.030 (4)	0.039 (4)	0.038 (4)	0.007 (3)	0.004 (3)	−0.004 (3)
C21	0.049 (4)	0.037 (4)	0.037 (4)	0.010 (3)	0.003 (4)	0.000 (3)
C22	0.047 (4)	0.043 (4)	0.037 (4)	0.007 (3)	0.004 (3)	−0.013 (4)
N1	0.033 (3)	0.025 (3)	0.041 (4)	−0.006 (2)	0.006 (3)	0.001 (3)
N2	0.029 (3)	0.011 (2)	0.024 (3)	0.002 (2)	0.005 (2)	−0.003 (2)
N3	0.022 (2)	0.011 (2)	0.026 (3)	−0.0013 (19)	0.005 (2)	0.001 (2)
N4	0.038 (3)	0.036 (3)	0.048 (4)	0.002 (2)	0.000 (3)	−0.003 (3)
O1	0.031 (2)	0.026 (2)	0.039 (2)	0.0052 (19)	0.0013 (19)	−0.006 (2)
O2	0.029 (2)	0.020 (2)	0.036 (3)	−0.0042 (16)	0.0006 (19)	−0.0048 (19)
O3	0.065 (3)	0.023 (2)	0.025 (2)	0.002 (2)	0.002 (2)	0.001 (2)
O4	0.0249 (19)	0.0142 (18)	0.028 (2)	0.0021 (17)	0.0053 (17)	0.0024 (19)
O5	0.045 (2)	0.017 (2)	0.021 (2)	0.0056 (17)	0.0079 (19)	−0.0030 (18)
O6	0.030 (2)	0.021 (2)	0.027 (2)	−0.0013 (18)	0.0064 (19)	0.006 (2)
O7	0.046 (3)	0.019 (2)	0.049 (3)	−0.005 (2)	0.019 (2)	−0.007 (2)
O8	0.066 (3)	0.035 (3)	0.106 (5)	−0.012 (2)	0.056 (3)	0.005 (3)
S1	0.0298 (8)	0.0166 (7)	0.0269 (9)	0.0041 (6)	0.0063 (7)	−0.0020 (6)
Cu1	0.0252 (3)	0.0066 (3)	0.0273 (4)	0.0008 (3)	0.0082 (3)	−0.0005 (3)

### Geometric parameters (Å, °)

**Table:**

C1—C6	1.382 (7)	C15—H15	0.9300
C1—C2	1.391 (7)	C16—N3	1.334 (6)
C1—S1	1.787 (5)	C16—C17	1.385 (7)
C2—C3	1.401 (6)	C16—H16	0.9300
C2—H2	0.9300	C17—H17	0.9300
C3—C4	1.373 (7)	C18—N4	1.335 (8)
C3—C7	1.525 (7)	C18—C19	1.389 (9)
C4—C5	1.398 (7)	C18—H18	0.9300
C4—N1	1.475 (7)	C19—C20	1.386 (9)
C5—C6	1.386 (7)	C19—H19	0.9300
C5—H5	0.9300	C20—C21	1.375 (8)
C6—H6	0.9300	C20—C20^i^	1.505 (11)
C7—O5	1.227 (6)	C21—C22	1.390 (8)
C7—O4	1.254 (6)	C21—H21	0.9300
C8—N2	1.341 (6)	C22—N4	1.342 (8)
C8—C9	1.381 (7)	C22—H22	0.9300
C8—H8	0.9300	N1—O8	1.216 (6)
C9—C10	1.388 (7)	N1—O7	1.219 (6)
C9—H9	0.9300	O1—S1	1.454 (4)
C10—C11	1.398 (7)	O2—S1	1.448 (4)
C10—C13	1.479 (6)	O3—S1	1.432 (4)
C11—C12	1.366 (6)	O6—H1A	0.83 (5)
C11—H11	0.9300	O6—H1B	0.86 (5)
C12—N2	1.339 (6)	Cu1—N2	1.986 (4)
C12—H12	0.9300	Cu1—N3^ii^	2.004 (4)
C13—C14	1.384 (7)	Cu1—O2^iii^	2.565 (4)
C13—C17	1.397 (7)	Cu1—O4	1.969 (3)
C14—C15	1.374 (6)	Cu1—O5^iv^	2.299 (4)
C14—H14	0.9300	Cu1—O6	2.032 (4)
C15—N3	1.337 (6)		
			
C6—C1—C2	119.9 (5)	N4—C18—C19	123.8 (7)
C6—C1—S1	121.2 (4)	N4—C18—H18	118.1
C2—C1—S1	118.9 (4)	C19—C18—H18	118.1
C1—C2—C3	121.4 (5)	C20—C19—C18	119.9 (7)
C1—C2—H2	119.3	C20—C19—H19	120.0
C3—C2—H2	119.3	C18—C19—H19	120.0
C4—C3—C2	116.7 (5)	C21—C20—C19	116.2 (6)
C4—C3—C7	126.0 (5)	C21—C20—C20^i^	121.8 (8)
C2—C3—C7	117.3 (5)	C19—C20—C20^i^	122.0 (8)
C3—C4—C5	123.6 (5)	C20—C21—C22	120.9 (6)
C3—C4—N1	119.4 (5)	C20—C21—H21	119.5
C5—C4—N1	116.9 (5)	C22—C21—H21	119.5
C6—C5—C4	117.9 (5)	N4—C22—C21	122.9 (6)
C6—C5—H5	121.0	N4—C22—H22	118.6
C4—C5—H5	121.0	C21—C22—H22	118.6
C1—C6—C5	120.5 (5)	O8—N1—O7	122.2 (5)
C1—C6—H6	119.8	O8—N1—C4	118.4 (5)
C5—C6—H6	119.8	O7—N1—C4	119.4 (5)
O5—C7—O4	128.8 (5)	C12—N2—C8	117.4 (4)
O5—C7—C3	117.5 (5)	C12—N2—Cu1	120.8 (4)
O4—C7—C3	113.6 (5)	C8—N2—Cu1	121.7 (4)
N2—C8—C9	122.5 (5)	C16—N3—C15	117.9 (4)
N2—C8—H8	118.7	C16—N3—Cu1^v^	123.2 (4)
C9—C8—H8	118.7	C15—N3—Cu1^v^	118.8 (4)
C8—C9—C10	119.9 (5)	C18—N4—C22	116.2 (6)
C8—C9—H9	120.0	C7—O4—Cu1	126.4 (3)
C10—C9—H9	120.0	C7—O5—Cu1^iv^	168.9 (4)
C9—C10—C11	117.2 (4)	Cu1—O6—H1A	105 (4)
C9—C10—C13	121.5 (5)	Cu1—O6—H1B	115 (4)
C11—C10—C13	121.4 (5)	H1A—O6—H1B	122 (5)
C12—C11—C10	119.3 (5)	O3—S1—O2	113.9 (2)
C12—C11—H11	120.4	O3—S1—O1	114.8 (2)
C10—C11—H11	120.4	O2—S1—O1	111.4 (2)
N2—C12—C11	123.7 (5)	O3—S1—C1	105.8 (3)
N2—C12—H12	118.1	O2—S1—C1	105.0 (2)
C11—C12—H12	118.1	O1—S1—C1	104.9 (2)
C14—C13—C17	117.7 (5)	O4—Cu1—N2	92.84 (16)
C14—C13—C10	121.1 (5)	O4—Cu1—N3^ii^	86.36 (16)
C17—C13—C10	121.1 (5)	N2—Cu1—N3^ii^	177.66 (18)
C15—C14—C13	119.9 (5)	O4—Cu1—O6	160.42 (16)
C15—C14—H14	120.1	N2—Cu1—O6	90.09 (17)
C13—C14—H14	120.1	N3^ii^—Cu1—O6	91.39 (17)
N3—C15—C14	122.6 (5)	O4—Cu1—O5^iv^	111.89 (14)
N3—C15—H15	118.7	N2—Cu1—O5^iv^	85.92 (16)
C14—C15—H15	118.7	N3^ii^—Cu1—O5^iv^	92.34 (16)
N3—C16—C17	123.2 (5)	O6—Cu1—O5^iv^	87.62 (15)
N3—C16—H16	118.4	O2^iii^—Cu1—N2	85.20 (17)
C17—C16—H16	118.4	O2^iii^—Cu1—N3^ii^	96.74 (17)
C16—C17—C13	118.5 (5)	O2^iii^—Cu1—O4	75.35 (13)
C16—C17—H17	120.7	O2^iii^—Cu1—O5^iv^	168.81 (12)
C13—C17—H17	120.7	O2^iii^—Cu1—O6	85.63 (13)
			
C6—C1—C2—C3	−1.2 (8)	C20^i^—C20—C21—C22	179.5 (7)
S1—C1—C2—C3	179.4 (4)	C20—C21—C22—N4	1.7 (10)
C1—C2—C3—C4	1.4 (8)	C3—C4—N1—O8	−177.7 (6)
C1—C2—C3—C7	−177.9 (5)	C5—C4—N1—O8	0.9 (8)
C2—C3—C4—C5	0.1 (9)	C3—C4—N1—O7	3.5 (8)
C7—C3—C4—C5	179.2 (5)	C5—C4—N1—O7	−178.0 (5)
C2—C3—C4—N1	178.5 (5)	C11—C12—N2—C8	1.0 (8)
C7—C3—C4—N1	−2.3 (9)	C11—C12—N2—Cu1	−176.6 (4)
C3—C4—C5—C6	−1.7 (9)	C9—C8—N2—C12	0.5 (8)
N1—C4—C5—C6	179.8 (5)	C9—C8—N2—Cu1	178.1 (4)
C2—C1—C6—C5	−0.5 (9)	C17—C16—N3—C15	−0.4 (8)
S1—C1—C6—C5	178.9 (5)	C17—C16—N3—Cu1^v^	179.2 (4)
C4—C5—C6—C1	1.9 (9)	C14—C15—N3—C16	1.6 (8)
C4—C3—C7—O5	95.3 (7)	C14—C15—N3—Cu1^v^	−178.0 (4)
C2—C3—C7—O5	−85.6 (6)	C19—C18—N4—C22	−3.8 (10)
C4—C3—C7—O4	−88.7 (7)	C21—C22—N4—C18	0.7 (9)
C2—C3—C7—O4	90.4 (6)	O5—C7—O4—Cu1	26.7 (7)
N2—C8—C9—C10	−1.5 (9)	C3—C7—O4—Cu1	−148.7 (3)
C8—C9—C10—C11	0.9 (8)	O4—C7—O5—Cu1^iv^	−29 (2)
C8—C9—C10—C13	179.0 (5)	C3—C7—O5—Cu1^iv^	146.5 (15)
C9—C10—C11—C12	0.6 (8)	C6—C1—S1—O3	−130.7 (5)
C13—C10—C11—C12	−177.6 (5)	C2—C1—S1—O3	48.7 (5)
C10—C11—C12—N2	−1.6 (8)	C6—C1—S1—O2	108.6 (5)
C9—C10—C13—C14	−35.0 (8)	C2—C1—S1—O2	−72.0 (5)
C11—C10—C13—C14	143.0 (5)	C6—C1—S1—O1	−9.0 (5)
C9—C10—C13—C17	147.2 (5)	C2—C1—S1—O1	170.5 (4)
C11—C10—C13—C17	−34.7 (8)	C7—O4—Cu1—N2	−106.1 (4)
C17—C13—C14—C15	2.0 (8)	C7—O4—Cu1—N3^ii^	71.7 (4)
C10—C13—C14—C15	−175.8 (5)	C7—O4—Cu1—O6	155.6 (5)
C13—C14—C15—N3	−2.5 (8)	C7—O4—Cu1—O5^iv^	−19.4 (4)
N3—C16—C17—C13	0.1 (8)	C12—N2—Cu1—O4	153.9 (4)
C14—C13—C17—C16	−0.9 (8)	C8—N2—Cu1—O4	−23.6 (4)
C10—C13—C17—C16	176.9 (5)	C12—N2—Cu1—O6	−45.4 (4)
N4—C18—C19—C20	4.6 (11)	C8—N2—Cu1—O6	137.1 (4)
C18—C19—C20—C21	−2.0 (10)	C12—N2—Cu1—O5^iv^	42.2 (4)
C18—C19—C20—C20^i^	177.6 (7)	C8—N2—Cu1—O5^iv^	−135.3 (4)
C19—C20—C21—C22	−0.9 (10)		

Symmetry codes: (i) −*x*, −*y*+1, −*z*+2; (ii) *x*, *y*+1, *z*; (iii) −*x*+1, *y*−1/2, −*z*+3/2; (iv) −*x*+1, −*y*+1, −*z*+1; (v) *x*, *y*−1, *z*.

### Hydrogen-bond geometry (Å, °)

**Table:**

*D*—H···*A*	*D*—H	H···*A*	*D*···*A*	*D*—H···*A*
O6—H1A···O1^iii^	0.83 (5)	1.94 (5)	2.758 (5)	171 (6)
O6—H1B···N4^vi^	0.86 (5)	2.00 (5)	2.801 (6)	156 (5)

Symmetry codes: (iii) −*x*+1, *y*−1/2, −*z*+3/2; (vi) −*x*+1, *y*+1/2, −*z*+3/2.
